# Molecular mechanisms underlying resistance to androgen deprivation therapy in prostate cancer

**DOI:** 10.18632/oncotarget.10901

**Published:** 2016-07-28

**Authors:** Kristine M. Wadosky, Shahriar Koochekpour

**Affiliations:** ^1^ Department of Cancer Genetics, Center for Genetics and Pharmacology, Roswell Park Cancer Institute, Buffalo, NY, USA; ^2^ Department of Urology, Roswell Park Cancer Institute, Buffalo, NY, USA

**Keywords:** prostate cancer, androgen receptor, castrate-recurrent, ADT, splice variant

## Abstract

Prostate cancer (PCa) is the most widely diagnosed male cancer in the Western World and while low- and intermediate-risk PCa patients have a variety of treatment options, metastatic patients are limited to androgen deprivation therapy (ADT). This treatment paradigm has been in place for 75 years due to the unique role of androgens in promoting growth of prostatic epithelial cells *via* the transcription factor androgen receptor (AR) and downstream signaling pathways. Within 2 to 3 years of ADT, disease recurs—at which time, patients are considered to have castration-recurrent PCa (CR-PCa). A universal mechanism by which PCa becomes resistant to ADT has yet to be discovered. In this review article, we discuss underlying molecular mechanisms by which PCa evades ADT. Several major resistance pathways center on androgen signaling, including intratumoral and adrenal androgen production, AR-overexpression and amplification, expression of AR mutants, and constitutively-active AR splice variants. Other ADT resistance mechanisms, including activation of glucocorticoid receptor and impairment of DNA repair pathways are also discussed. New therapies have been approved for treatment of CR-PCa, but increase median survival by only 2-8 months. We discuss possible mechanisms of resistance to these new ADT agents. Finally, the practicality of the application of “precision oncology” to this continuing challenge of therapy resistance in metastatic or CR-PCa is examined. Empirical validation and clinical-based evidence are definitely needed to prove the superiority of “precision” treatment in providing a more targeted approach and curative therapies over the existing practices that are based on biological “cause-and-effect” relationship.

Prostate cancer (PCa) is the most common male cancer in the Americas, Caribbean, Western and Northern Europe, Australia, New Zealand, and several countries in sub-Saharan Africa [1]. The American Cancer Society estimated that 220800 PCa cases were diagnosed in the United States in 2015 [2], while 416700 new cases in Europe were estimated for 2012 [3]. Despite its prevalence, PCa is not the leading cause of cancer-related death in most of these regions, with lung cancer foremost among mortality rates for North America, Western Europe, Australia, New Zealand, and South Africa [1]. Global trends show that while the incidence of PCa has risen, PCa-related mortality has either decreased or remained unchanged in most countries studied [1]. These figures are attributed largely to the advent of the serum prostate specific antigen (PSA) test in the mid-1980s and early-1990s, a screening method far superior to the digital rectal examination (DRE) [4]. Currently, there is ongoing debate about whether population-based PSA screening is appropriate, the details about which have been extensively reviewed [5–12]. Nevertheless, serum PSA continues to be a key metric in PCa diagnosis, staging, and treatment decisions.

## DIAGNOSIS OF PROSTATE CANCER

Serum PSA and DRE are used in combination to determine if a biopsy should be performed, usually *via* the transrectal ultrasound-guided (TRUS) method [7]. Other imaging techniques, including magnetic resonance imaging (MRI), computed tomography (CT), and radionuclide bone scans can provide additional information for PCa staging [13]. Furthermore, the utility of positron emission tomography (PET) in PCa diagnosis remains under investigation [13, 14]. Despite the progress made in imaging technology, the mainstay of PCa diagnosis is histological examination. In 1966, Dr. Donald F. Gleason proposed a diagnostic grading system based on the morphological architecture of the tumor with emphasis on glandular structure [15]. Importantly, Gleason proposed that the two most common patterns be reported, with the most prevalent listed first and the next most prevalent listed second, where the final score is the addition of the two grades [15]. Along with Dr. George T. Mellinger, Gleason demonstrated the clinical relevance of this scoring technique in 1974 and it has been in use ever since [16, 17]. The intricate pathological details comprising the “Gleason system” and how it has evolved over the decades have been reviewed extensively [17–20] and are outside the scope of this article. But it is important to note that persistent issues with the Gleason system, despite multiple rounds of revisions, have induced the urological pathology community to introduce a new PCa grading system in 2015 [19, 20]. This new classification still makes use of the Gleason system, but groups Gleason scores into Grade Groups 1-5 that better reflect prognosis and simplifies diagnosis for patients; therefore, future PCa diagnoses will include both Gleason score and Grade Group [19, 20]. Since all basic and clinical research studies examined in this article pre-date this change, only Gleason score will be discussed.

## STAGING AND INITIAL TREATMENT OF LOCALIZED PROSTATE CANCER

Following diagnosis, PSA, Gleason score, and general tumor staging are used to assess overall prognosis [21, 22]. While specific risk stratification paradigms vary, cases are generally grouped into low-, intermediate-, or high-risk (Table 1) [7, 23–27]. Men with low-risk PCa comprise the majority of patients, reported by the American Cancer Society in 2015 to be 93% of all new cases [2]. Low-risk PCa is localized to the prostate with a Gleason score ≤ 6 and patients with this type of tumor tend to have low-volume disease and serum PSA ≤ 10 ng/mL (Table 1) [7, 23, 26, 28, 29]. There are a wide variety of treatment options for low-risk PCa, including radical prostatectomy, external beam radiotherapy, and brachytherapy (Table 1) [29, 30]. Cryotherapy and high intensity focused ultrasound can also be used in low-risk cases, but these therapies are less common (Table 1) [29, 30]. In addition, patients in this group may also elect for watchful waiting or active surveillance (Table 1). These alternatives are based on the idea that quality of life may be diminished by treatment more so than by the disease itself [31, 32]. While evidence shows that observation is advantageous for patients with life-expectancy of ≤ 10 years [31], there remains to be a definitive study in this risk group that shows any of the other treatment strategies to be superior [29, 30]. Therefore, choice of treatment for low-risk PCa is made on a case by case basis and is largely driven by a patient's personal preferences [29, 30].

**Table 1:** Prostate cancer diagnostic categories and initial treatment options

Stage	Clinical Characteristics	Treatment
Low-risk	Gleason score ≤ 6 and PSA ≤ 10 ng/mL and Organ confined, low volume	Active surveillance, watchful waiting, radical prostatectomy, radiotherapy, brachytherapy, cryotherapy, high intensity ultrasound
Intermediate-risk	Gleason score 7 or/and PSA 10-20 ng/mL or/and Organ confined or Regional metastases Low or high volume	Radical prostatectomy + radiotherapy, Brachytherapy + radiotherapy ± ADT
High-risk/Locally Advanced	Gleason score 8-10 or/and PSA > 20 ng/mL or/and Organ confined or Regional metastases High Volume	Radical prostatectomy + radiotherapy, Radical prostatectomy + ADT, Radiotherapy + ADT or Primary ADT
Advanced/Metastatic	Distant metastases	Primary ADT

Men who are diagnosed with intermediate-risk PCa are a more heterogeneous population than those in the low-risk group [21]. Typically, a patient is considered to have intermediate-risk PCa if he has at least one of the following clinical features: Gleason score 7, PSA 10 - 20 ng/mL, or regional metastases, such as to the pelvic lymph nodes or seminal vesicles (Table 1) [21, 26, 29]. As can be expected, the multitude of possible combinations complicates the staging of these PCa cases. No clinical trials have conclusively shown the advantage of any particular treatment paradigm for intermediate-risk patients, but a combined therapeutic approach is typically utilized (Table 1) [29, 30]. Often included in treatment scenarios for the intermediate-risk population is androgen deprivation therapy (ADT) (Table 1), a strategy in use for more than 70 years [33]. The goal of ADT is to deprive the PCa tumor of androgens, steroid hormones that drive prostate epithelial cell growth and proliferation [33]. The primary mechanism by which testosterone and dihydrotestosterone (DHT) promote PCa formation and growth is by activating androgen receptor (AR), a transcription factor to be discussed in following sections. In general, drugs used in ADT target either androgen production or AR activation and can be used alone or paired with one from the other category (combined androgen blockade (CAB)) [33]. ADT can be added to a treatment regimen before, concurrently, or after a neoadjuvant approach [26, 29].

In the most simplistic of paradigms, PCa cases are considered high-risk with a Gleason score 8-10, PSA ≥ 20 ng/mL, and indications of high tumor volume with or without regional metastases (Table 1) [27]. As with the intermediate-risk population, clinical characteristics of these PCa patients lack uniformity; thus, adherence to strict limits for each prognostic metric may not be possible for every case [26, 27]. Additional prognostic indicators, such as PSA velocity per year, PSA doubling time, and percentage of positive biopsy cores, have been incorporated into risk stratifications to attempt to pinpoint patients who need more aggressive treatment [21, 27].

There has yet to be a consensus on the absolute definition of high-risk PCa [27, 34]. Given the higher probability of treatment failure and increased metastatic potential in this population, early identification of these PCa patients is a priority [27, 34]. A biomarker that reliably predicts which patients are at the greatest risk would be a valuable tool, however one remains to be identified [21, 27, 34].

The initial treatment options for high-risk PCa patients include radical prostatectomy with lymph node dissection or radiotherapy with concurrent/adjuvant ADT (Table 1) [26, 34]. Evidence from available retrospective trials is not considered decisive enough for the clinical community to recommend one treatment over the other for high-risk cases [26, 34]. An obvious advantage of radical prostatectomy is that surgical results provide a conclusive pathological stage and identifies the extent of tumor invasion—indeed, surgical margins, whether positive or negative, can be used to guide future treatment decisions [35]. Often, evidence of lymph node involvement or positive surgical margins following radical prostatectomy prompts adjuvant treatment with either radiation or ADT [26, 34]. Ultimately, the decision between surgery and radiation for initial therapy lies with the patient and the expertise of his urologic oncology team [34]. Lastly, ADT can also be used in this population as a monotherapy, termed primary ADT (Table 1); however, the multimodal approaches described above are favored for high-risk patients without distant metastases [24, 26, 29, 36].

## INITIAL TREATMENT OF ADVANCED/METASTATIC PROSTATE CANCER

PCa patients with disease showing evidence of distant metastasis at diagnosis are considered the most advanced and treatment options for this group are limited (Table 1). The standard of care for advanced/metastatic PCa cases is long-term primary ADT, which includes surgical castration or medical castration with a luteinizing hormone releasing hormone (LHRH) agonist or CAB [26, 37]. Detailed mechanistic information about the drugs utilized in primary medical castration and CAB has been extensively reviewed by our group [33]. Disease response to ADT is substantial—where indications of metastatic disease, bone pain, and PSA levels decrease in the majority of patients within a short time [38]. Recent meta-analysis and data from multicenter trials have shown that addition of the chemotherapeutic agent docetaxel to ADT for treatment-naïve metastatic patients increases overall survival compared to ADT alone [39, 40]. While adverse events were reported in a higher percentage of patients who received ADT and docetaxel, the authors urge that long-term ADT with docetaxel be considered standard of care in initial treatment of men who are fit to receive chemotherapy [39–41]. At the time of this writing, these data have just been released—therefore it is unknown whether “chemohormonal” therapy for primary treatment of metastatic PCa will become accepted in practice or if the survival advantage will translate to a larger patient population.

Treatment options being confined to ADT for metastatic PCa patients is problematic because ADT is correlated with significant morbidity and decreased quality of life [42]. Castration has been associated with decreased bone mineral density and increased risk of fracture, effects that are especially significant since PCa preferentially metastasizes to bone [42, 43]. Skeletal muscle atrophy is another effect associated with ADT [42, 44, 45], but whether loss of muscle mass is accompanied by decreased muscle strength varies by study [44]. In addition to skeletal muscle, ADT is linked to adverse effects on cardiac muscle and has been associated with increased incidence of heart failure and myocardial infarction [42, 46, 47]. While some studies have shown that ADT increases cardiac-specific mortality in patients with cardiac risk factors [48, 49], others have shown no association [42, 46, 50, 51]; therefore, whether ADT is life-threatening for patients with preexisting cardiovascular disease remains to be determined [47]. Other cardiovascular disorders, including dyslipidemia, anemia, and stroke have also been associated with ADT [42]. Moreover, ADT has the potential to affect a patient's metabolism—for instance, PCa patients receiving ADT can have decreased insulin sensitivity, increased fasting glucose and insulin, and a greater incidence of diabetes [42, 52]. In addition, ADT has been shown to increase a patient's weight by about 2% and fat mass by about 10% within a 12-month period [52]. The potentially life-threatening side effects of ADT described above are joined by other complications including loss of libido, decreased sexual function, gynecomastia, hot flashes, and fatigue [42]. Despite the substantial adverse effects of ADT on cardiovascular and overall health, the American Heart Association, American Cancer Society, and American Urological Association agree that the benefits of ADT outweigh the risks for metastatic PCa patients [47].

## CASTRATE-RECURRENT PROSTATE CANCER

Despite increasing overall survival for metastatic PCa patients, ADT is not considered curative since only 5-10% of patients survive > 10 years after the start of treatment [38, 53]. Continuous ADT merely suppresses symptoms and indicators of disease for 2-3 years [38]. After which, a patient has recurred if PSA levels have increased on two separate occasions between 1 to 3 weeks apart, even after adjustments of hormonal therapy [26, 37, 54]. Rising PSA levels may be accompanied by evidence of primary and/or metastatic lesion progression, despite castrate levels of testosterone at < 50 ng/dl [26, 37, 54]. These patients are considered to have castration-recurrent PCa (CR-PCa) and are expected to survive for only 16-18 months following relapse [38]. This disease state has previously been known as androgen-independent, hormone-resistant, or hormone-refractory, but these terms have been abandoned after overwhelming evidence in recent years has shown that CR-PCa tumors remain dependent on androgen signaling and AR-dependent activity [38, 55]. Indeed, two drugs that have recently been FDA-approved for treatment of CR-PCa include abiraterone and enzalutamide, which target androgen biosynthesis and AR, respectively [33]. While the available therapies for CR-PCa, abiraterone, enzalutamide, docetaxel and cabazitaxel, statistically extend median survival, they do so by only 2-8 months (Table 2) [56–63]. To date, a universal mechanism by which PCa becomes resistant to ADT has yet to be discovered. Instead, data suggest that there are several modes by which PCa cells continue to survive under castration. As discussed in detail below, many of the major resistance pathways center on AR [64], but others depend on other mechanisms (e.g. glucocorticoid receptor (GR) [65], DNA repair [66]). It is the hope of the clinical and research community that understanding the biological basis for PCa resistance to ADT will lead to a curative therapy that can be used in junction with or in place of hormonal modulation.

**Table 2:** Overall survival increase following current CR-PCa therapies

Drug	Control Group	Treatment Stage	Increased survival	Reference
Abiraterone + prednisone	*vs*. prednisone alone	Post-chemotherapy	3.9 months	[56]
		Pre-chemotherapy	8.2 months	[57]
Enzalutamide	*vs*. placebo	Post-chemotherapy	4.8 months	[59]
		Pre-chemotherapy	2.2 months	[58]
Docetaxel + prednisone	*vs*. mitoxantrone + prednisone	n/a	2.9 months	[60, 62]
Docetaxel + estramustine	*vs*. mitoxantrone + prednisone	n/a	1.9 months	[61]
Cabazitaxel+ prednisone	*vs*. mitoxantrone + prednisone	Post-docetaxel	2.4 months	[63]

## MECHANISMS OF RESISTANCE TO ANDROGEN DEPRIVATION THERAPY

### Production of intratumoral and adrenal androgens

Neither surgical nor medical castration completely eliminates intratumoral androgens in PCa [67–69]. Tumor levels of DHT measured in PCa patients after 6 months of ADT show that DHT remains at 25% of original levels [67]. This study also found that intratumoral DHT levels do not significantly correlate with serum testosterone levels [67], an important finding since serum testosterone is one measure used by clinicians to assess ADT response [26]. Another study measuring intratumoral DHT in CR-PCa during ADT found that levels were decreased 91% after a median of 37 months of treatment compared to benign prostatic tissue from patients with localized PCa who did not undergo ADT [68]. There is also evidence to suggest that metastatic PCa tumors maintain androgen levels (ranging between 0.2 to 1.78 ng/g) despite ADT—where testosterone has been shown to be up to 4 times higher in metastatic compared to non-cancerous tissues [69]. In these studies, the absolute tissue levels of testosterone or DHT are above the threshold level of androgens required to induce proliferation of PCa cells cultured under androgen-deprived conditions [70, 71]. In addition, CR-PCa intratumoral androgen concentrations reported are also sufficient to bind to and activate AR, the requirements for which have been shown to be < 0.2 nM in multiple PCa cell lines [70, 71]. Therefore, despite substantial reduction in serum and intratumoral testosterone/DHT, the minimal concentration that persists is adequate to activate the molecular pathways that drive PCa growth.

Clearly, bilateral orchiectomy eliminates only testicular production of androgens, but modulation of the LHRH signaling axis in medical castration also primarily targets testicular androgen synthesis [33, 72]. While the majority of androgen hormones are produced in the testes, about 10% of androgen synthesis occurs in the adrenal glands and peripheral tissues [72]. The corticotropin-releasing hormone-adrenocorticotropic hormone (CRH-ACTH) signaling axis, which remains active during ADT, induces the production of androstenedione and dehydroepiandrosterone (DHEA) by the adrenal glands [72]. Consequently, maintenance of adrenal androgens, shown to decrease by only 60% during ADT [67], is considered a mechanism by which PCa becomes resistant to therapy. Development of inhibitors of cytochrome P450 17 (CYP17), an enzyme involved in androgen biosynthesis *via* multiple pathways and precursors [73], was established to directly inhibit androgen production [72]. The result of this work produced abiraterone [56, 57], which is FDA-approved for use in CR-PCa, but has produced minimal results in prolonging survival (Table 2). Recent analysis has shown that while DHEA and its sulfated form are decreased following treatment with abiraterone and the pan-CYP inhibitor ketoconazole, these drugs were unable to reduce serum DHEA and DHEA-S to levels below 20 μg/dL [74]. Membrane transport of DHEA-S into peripheral cells is facilitated by organic anion transporting polypeptides (OATPs) [75] and it has been shown that the expression of OATP1A2 increases in response to androgen deprivation in LNCaP and 22Rv1 PCa cell lines [76]. In addition, knockdown of OATP1A2 abrogated DHEA-stimulated proliferation of LNCaP cells, illustrating that DHEA's effect depends on this transmembrane transporter protein [76]. These data suggest that ADT may promote influx of steroid precursors that enable testosterone synthesis within the PCa cell [74, 77]. In addition to stimulating DHEA uptake by PCa cells *in vitro*, ADT has been shown to increase *in vivo* expression of enzymes required for testosterone synthesis in CR-PCa tumor xenografts and tissue samples [78, 79]. In addition, experiments culturing excised CR-PCa patient tumor samples *ex vivo* with steroid precursors have shown that these tissues are capable of producing androgen hormones, as identified *via* LC-MS analysis, including testosterone and others upstream in the biosynthesis pathway [79, 80]. Cholesterol also contributes to *de novo* androgen production in PCa cells under androgen-deprived conditions, since cholesterol is the primary upstream precursor in steroidogenesis [81]. Castrate-recurrent cell lines DU145, PC3, and LNCaP C81 express higher levels of enzymes involved in steroidogenesis, including scavenger receptor type B1, steroidogenic acute regulatory protein, cytochrome P450 cholesterol side chain cleavage, and 3β-hydroxysteroid dehydrogenase, at the mRNA and protein levels compared to androgen-dependent LNCaP C33 and normal prostate epithelial cells [82]. When either C33 or C81 cell lines were cultured with exogenous cholesterol in serum-free medium, castrate-recurrent C81 produced a significantly increased amount of testosterone compared to androgen-dependent C33, as measured by a radioimmunoassay of the conditioned media [82]. In the LNCaP xenograft model, it was shown that CR-PCa tumors (35 days post-castration) had significantly increased amount of synthesized cholesterol compared to those at pre-castration or 8 days post-castration (PSA nadir) [83]. In addition, enzymes required for cholesterol influx, efflux, synthesis, and metabolism were increased at the protein level in CR-PCa tumors compared to pre-castration and 8 days post-castration tumors [83]. These studies in castrate-recurrent cell lines and xenograft model of CR-PCa progression show that both increased production of cholesterol and conversion of cholesterol to testosterone contribute to resistance to androgen deprivation [82, 83]. Together, maintenance of low levels of intratumoral testosterone/DHT, continued production of the adrenal androgen DHEA, and *de novo* synthesis of testosterone from cholesterol all contribute to development of CR-PCa. Additional CYP17 inhibitors currently in clinical trials for the treatment of CR-PCa include galeterone, VT-464, and CFG920 [33]. However, the clinical effectiveness of these new CYP17 inhibitors and their ability to inhibit adrenal DHEA production and intratumoral androgen synthesis remain to be determined.

### Resistance mechanisms related to androgen receptor

AR is central to PCa biology *via* its role as mediator of growth and proliferation of prostatic epithelial cells in response to testosterone [33]. Consequently, research on understanding mechanisms of ADT resistance in PCa has focused largely on AR [84]. Mechanisms governing development of CR-PCa has been linked to aberrant AR signaling at the gene, transcript, and protein levels [84].

### Increased expression of androgen receptor

**Figure 1:**
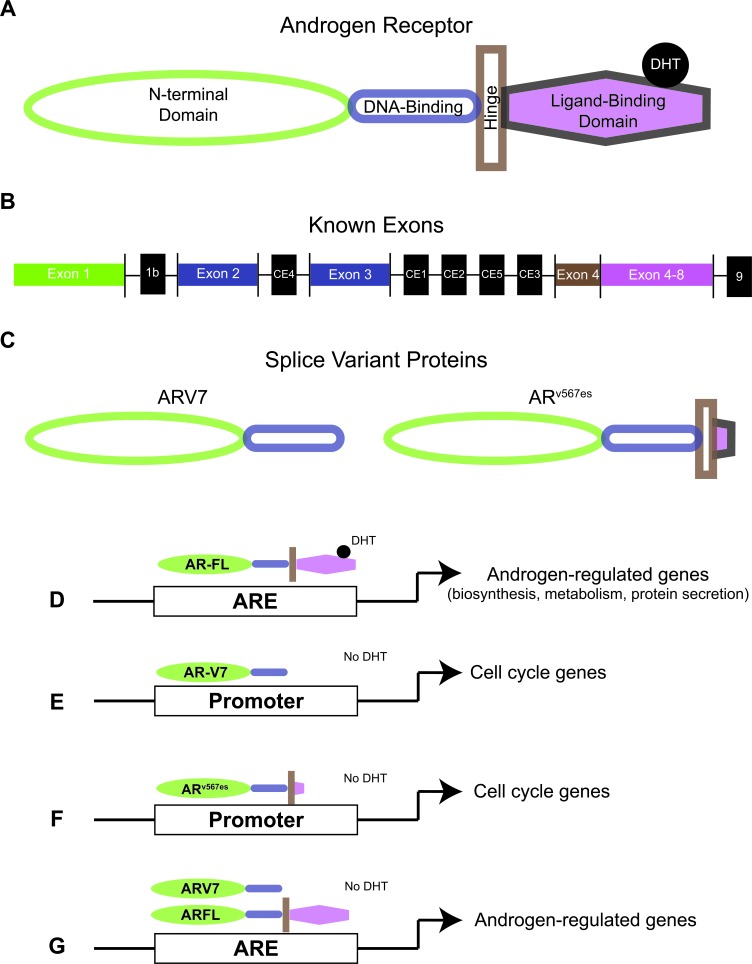
Androgen receptor and androgen receptor splice variant protein structure and activity (A) Protein structure of full-length AR. (B) AR mRNA exon structure. Exons that code for full length AR are in color (corresponding to protein structure) and exons that only code for splice variants are in black (C) Protein structures of AR splice variants ARV7 and ARv567es. (D) Full length AR activates androgen regulated genes in the presence of DHT. (E) ARV7 activates expression of cell cycle genes without DHT. (F) ARv567es activates expression of cell cycle genes without DHT. (G) ARV7 and full length AR co-occupy promoters of androgen regulated genes and activate transcription Without DHT. AR: Androgen receptor; DHT: Dihydrotestosterone.

Located at Xq11-12, AR is 90 kb in length with 8 exons that produces a 110 kDa protein with 919 amino acids [33]. The protein structure of AR coincides with that of other nuclear receptors, containing N-terminal (NTD), DNA-binding (DBD), hinge, and ligand-binding (LBD) domains (Figure 1A) [33]. The cloning of AR was published simultaneously by Dr. Shutsung Liao's and Dr. Elizabeth Wilson's research groups in 1988 [85, 86]. After identifying the location of AR, it was hypothesized that development of therapy resistance in PCa is associated with alternations in that region of the X chromosome. Indeed, several studies showed that AR amplification occurs in CR-PCa, with this genomic aberration taking place in 20-30% of patients, depending on the study [87–90]. In addition, paired samples from primary and recurrent tumors from the same patient show that AR amplification, as measured by fluorescence in situ hybridization (FISH), occurs when the tumor transitions to a resistant state [87]. FISH studies using tissue microarrays showed that AR amplification is present in only 2% of the primary PCa tumor and none of the benign prostatic hyperplasia (BPH) samples, compared to 23.4% of CR-PCa tumors [88]. Finally, RT-PCR analysis confirmed that AR amplification is indeed reflected at the message level, where AR mRNA expression in CR-PCa tumors with AR amplification was increased 2-fold compared to CR-PCa tumors without AR amplification [90]. In addition, expression microarray analysis using xenograft tumor samples of isogenic androgen-sensitive and recurrent pairs showed that increased AR was the only gene expression alteration that was consistent over all 7 different CR-PCa xenograft models [91].

In line with AR amplification and increased mRNA expression, elevated AR protein levels are also linked to CR-PCa. Protein expression of AR is increased in recurrent tumor samples compared to paired androgen-sensitive samples in multiple isogenic tumor xenograft models [91, 92]. Specifically, in a CWR22 xenograft that models the transition from androgen-sensitive to recurrent growth, AR protein was gradually decreased during the 120-day castration period and then reestablished in recurrent tumors [92]. While these observations of AR protein levels are a direct result of gene amplification and elevated mRNA expression, increased protein half-life can also contribute to augmented AR protein levels in CR-PCa [93]. Stabilization of AR protein is associated with alterations in both its post-translational modifications [94] and interaction with protein chaperones, such as those in the heat shock protein (HSP) family [95]. However, while several *in vitro* studies suggest that pathways involved in AR proteostasis could be involved in ADT resistance [96–99], one has yet to emerge that is definitely linked to CR-PCa.

Together, these data reflect resistance mechanisms that increase AR gene, transcript, and protein and illustrate the complex regulation of AR levels in CR-PCa. Increased AR expression not only creates a molecular environment that is hypersensitive to androgen stimulation [100], but is also capable of converting AR antagonists, such as bicalutamide and cyproterone acetate, to agonists [91]. This phenomenon is being utilized clinically in CR-PCa patients by withdrawing anti-androgens from the ADT treatment regimen [26], a strategy that has been shown to decrease PSA and increase progression-free survival [101]. This scenario demonstrates the flexibility that is required in the treatment of PCa as the molecular landscape of the tumor changes in response to long-term non-curative ADT.

### Androgen receptor mutants

The study of AR in PCa has identified AR's LBD (Figure 1A and 1B) as the principal protein region governing resistance. Only a few years after the cloning of AR, a LBD point mutation was identified in the LNCaP cell line [102], derived from the lymph node of a patient with metastatic PCa [103]. This mutation causes an amino acid substitution at position 878 from threonine (T) to alanine (A) (T878A) (Table 3) [102]. Ligand binding and activation of the AR T878A mutant occurs in response to androgens (testosterone, DHT, and DHEA), but also to non-androgen hormones (estradiol and progesterone), and AR antagonists (cyproterone acetate, flutamide, and nilutamide) (Table 3) [102, 104–106]. It has been shown that this mutation is also expressed in the cell lines MDA-PCa2a and MDA-PCa2b, established from the bone metastasis of an African American CR-PCa patient [107], and C4-2B, a highly tumorigenic cell line derived from LNCaP (Table 3) [108, 109]. AR T878A has been identified in tissues of CR-PCa patients, but not hormone-naïve patients, suggesting that this mutation occurs as a result of prolonged ADT [106, 110, 111]. The activation of the AR T878A mutant by the AR antagonist flutamide has led to the hypothesis that expression of this mutant is responsible to the beneficial effect of withdrawing anti-androgen therapy in CR-PCa patients. Indeed, it was found that this mutant was expressed in the tumors of CR-PCa patients whose PSA levels were greatly decreased by flutamide withdrawal [106].

**Table 3:** Androgen receptor mutants expressed in CR-PCa tumors and cell lines

Mutation	Aberrant Effect	Cell Line Expression	References
T878A	Activated by DHEA^a^, estradiol, progesterone, cyproterone acetate, flutamide, nilutamide	LNCaP, C4-2, MDA-PCa2a/b	[99, 101–108]
H875Y/T	Activated by DHEA, estradiol, progesterone, flutamide, nilutamide	22Rv1, CWR-R1	[99, 101–102]
W742C	Activated by bicalutamide	Long-term treatment of LNCaP with bicalutamide	[109]
L702H	Activated by glucocorticoids	MDA-PCa2a/b	[102, 104, 111–112]
F877L	Activated by enzalutamide, ARN-509	Long-term treatment of LNCaP with enzalutamide, ARN-509	[154]

a DHEA: Dehydroepiandrosterone

Another mutation located in the AR LBD has been discovered in LNCaP cells that is induced by prolonged treatment with bicalutamide [110]. This mutation is located at codon 742 and causes a tryptophan (W) to cysteine (C) amino acid substitution (W742C) (Table 3) [110]. AR W742C, also containing the T878A mutation, is activated by the AR antagonist bicalutamide (Table 3) [110]. Interestingly, the W742C/T878A mutant retains its ability to be inhibited by flutamide [110], in stark comparison to the T878A single mutant which is activated by flutamide (Table 3) [101, 103]. The CWR22 cell line, as well as the recurrent cell lines 22Rv1 and CWR-R1 derived from serial transplantation of CWR22 xenografts, also harbor an AR LBD mutation (Table 3) [109]. This mutation is located at position 875 and causes the amino acid substitution from histidine (H) to either threonine (Y) or tyrosine (T) (H875Y/T) [105]. As with AR T878A, AR H875Y/T is activated by the hormones DHEA, estradiol, and progesterone and the AR antagonist flutamide [104]. Finally, a mutation at position 702 of AR, causing an amino acid substitution of leucine (L) to histidine (H) (L702H), is expressed in the MDA-PCa2a/b cell lines, which also contain the T878A mutation as discussed above (Table 3). This L702H/T878A double mutant is activated by multiple glucocorticoid hormones [107, 112], but inhibited by both bicalutamide and flutamide [113]. Since glucocorticoid hormones can be increased during the clinical course of ADT and are often administered in conjunction with androgen-targeted therapies [33], the AR L702H mutation likely plays a significant role in development of CR-PCa in patients who express this mutation.

Recent estimates show that AR T878A, H875T/Y, W742C, and L702H are present in 15-20% of CR-PCa samples [55, 114, 115], establishing the region of AR that codes for the LBD as a hotspot for mutation. Recently, circulating free DNA from CR-PCa patients has been shown to contain genomic DNA with the AR mutations described above, showing that detection of these point mutations by sequencing could possibly be a biomarker for patients at risk for developing CR-PCa [116]. These data demonstrate that AR mutants are significant contributors to therapy resistance, especially since they are not only capable of being activated by adrenal androgens and other steroid hormones, but also by the AR antagonists meant to inhibit them (Table 3).

### Androgen receptor phosphorylation

Post-translational modification of AR by phosphorylation plays a role in its protein stability, transcriptional activity, and nuclear localization [94, 117]. Phosphorylation of AR can occur at serine, threonine, or tyrosine residues and 16 in total are known to be phosphorylated, most of which are located in the NTD [94]. Comprehensive analysis of each phosphorylated residue and its effect on AR function has been reviewed elsewhere [94, 117]. Many of these AR phosphorylation events have been studied in androgen-dependent cell lines under androgen-containing conditions, therefore it is unclear how these phosphorylated residues contribute to AR-dependent development of resistance to androgen-deprivation and CR-PCa [94]. Here we focus on AR phosphorylation that has been linked to PCa resistance to ADT both in cell culture models of recurrence and in CR-PCa patients. For these particular phosphorylation sites, growth factor stimulation plays a role in activating AR *via* phosphorylation, thereby promoting continued PCa cell proliferation under androgen-deprived conditions. Specifically, epidermal growth factor (EGF) treatment of the castrate-recurrent CWR-R1 cell line cultured in low-androgen conditions promotes phosphorylation of AR at serine 515 (Ser515) and serine 578 (Ser578), mitogen activated protein kinase (MAPK) and protein kinase C (PKC) consensus sites, respectively [118]. AR Ser515 and Ser578 phosphorylation promote AR transcriptional activity, since PSA-luciferase activity was inhibited in CWR-R1 cells cultured in low-androgen conditions when either or both serine residues were mutated to alanine, an amino acid that cannot be phosphorylated [118]. Phosphoinositide 3-kinase (PI3K)/Akt signaling has been implicated by Lin et al. in PCa progression [119]. Overexpression of Akt in low-passage LNCaP inhibited AR transcriptional activity, as measured by a MMTV-luciferase assay and PSA immunoblot, but activated AR transcriptional activity in high-passage LNCaP [119]. These data suggest that Akt-dependent activation of AR may occur in models of advanced PCa; however, this relationship between Akt and AR was not assessed in LNCaP-derived recurrent cell lines (such as C4-2 and C4-2B) [109, 119, 120]. In the same study, it was shown that expression of a serine 213 (Ser213) to alanine AR mutant in the COS1 kidney fibroblast cell line decreased insulin-like growth factor 1 (IGF1)-dependent serine phosphorylation of AR [119]. Since IGF1 is a growth factor that activates PI3K/Akt signaling, these data suggest that AR Ser213 phosphorylation occurs downstream of PI3K/Akt activation, but IGF1-dependent AR phosphorylation at Ser213 was not assessed in PCa cells in this study [119]. More recently, immunoblot using a phospho-specific antibody for AR Ser213 has shown that phosphorylation of this residue occurs in LNCaP and castration-recurrent cell lines LNCaP95, 22Rv1, and VCaP [121]. However, androgen-deprivation and concomitant treatment with the second-generation AR antagonist enzalutamide showed cell line-specific effects on AR Ser213 phosphorylation [121]. For instance, ADT increased AR Ser213 phosphorylation in LNCaP and LNCaP95, decreased AR Ser213 phosphorylation in 22Rv1, and had no effect on AR Ser213 phosphorylation in VCaP [121]. Since 22Rv1 and VCaP are Akt negative cell lines, these data suggest both that androgen deprivation promotes Ser213 phosphorylation of AR and that Akt is the primary kinase responsible for this phosphorylation [121]. When phospho-Akt and phospho-AR Ser213 were assessed by immunohistochemistry in matched hormone-naïve and CR-PCa tumors, increases in both were found to correlate with decreased disease-specific survival [122]. In a later study, the same research group used a panel of phospho-specific antibodies to assess the expression of phosphorylated AR at serines 94 (Ser94), 308 (Ser308), 650 (Ser650), and 791 (Ser791) in matched hormone-naïve and castrate-recurrent PCa tumors [123]. No correlations were found for phospho-AR at either Ser94 or Ser650, but surprisingly increased phospho-AR Ser308 was associated with longer time to disease-specific death and increased Ser791 was associated with longer time to recurrence [123]. These data suggest that while AR Ser213 phosphorylation appears to be associated with resistance to androgen deprivation and progression in PCa patients, phosphorylation of AR at either Ser308 or Ser791 may inhibit progression [122, 123]. Overall, despite the proven effect of phosphorylation on AR expression and activity in PCa cells and other cell types [94, 117], a universal role of AR phosphorylation in recurrence of PCa following ADT has yet to be determined. It is possible that regulation of AR by phosphorylation may only be secondary to other AR-dependent molecular mechanisms of castration resistance in PCa, such as increased expression of AR, expression of AR mutants, and AR splice variants.

### Androgen receptor splice variants: methods of discovery

As discussed in the previous section, AR mutations clinically relevant to therapy resistance are located in the LBD, demonstrating that alternations in this domain can drive CR-PCa [55]. Further support for the LBD's importance in CR-PCa is shown by several lines of evidence over the past decade of the existence of constitutively-active AR variants that lack the LBD [124, 125]. Using 3′ rapid amplification of cDNA ends (RACE) with a forward primer anchored at AR exon 1, two distinct transcripts that contained exon 1, 2, or 3 and a novel nucleotide sequence (cryptic exon 4 [CE4]) were discovered [126]. Named AR1/2/2b and AR1/2/3/2b, these transcripts code for the NTD and a partial or full DBD, respectively (Table 4) [126]—allowing the putative truncated AR proteins to bind to DNA and interact with co-receptors irrespective of androgen status. Expression of AR1/2/2b or AR1/2/3/2b in the AR-negative PCa cell line DU145 showed that MMTV-luciferase activity was equally activated despite treatment with mibolerone, a potent steroid ligand of AR, providing evidence of their constitutive activity [126]. Several studies completed soon after the publication of Dehm et al. confirmed the discovery of AR splice variants [126]. Hu et al. identified CE 1-4 when searching for AR intronic sequences in the human expressed sequence database and discovered AR variants ARV 1-7 that all lacked the LBD (Figure 1B) [127]. The AR transcripts ARV3 and ARV4 had identical sequences to AR1/2/2b and AR1/2/3/2b; therefore, new variants identified were ARV1, ARV2, ARV5, ARV6, and ARV7, where ARV5 and ARV6 differed only by short unique 3′ sequences (Table 4) [126, 127]. Since ARV7 was the most consistently expressed in a panel of CR-PCa cell lines, the authors generated an ARV7-specific antibody against the putative unique peptide sequence coded for by CE3 [127]. Immunoblot using the ARV7 antibody in LNCaP, VCaP, 22Rv1, and PC3 cells resulted in protein bands at 75kDa, the predicted molecular weight of ARV7, in VCaP and 22Rv1 only [127]. Importantly, these data showed for the first time that an AR variant transcript is fully translated (Figure 1C) [127]. A third independent study used 3′ RACE with a primer anchored in AR exon 1 and identified three variant AR transcripts [128]. As shown in Table 4, these three variants, named AR3, AR4, and AR5, were identical to the previously published transcripts ARV7, ARV1, and AR1/2/3/2b/ARV4, respectively [126–128]. Guo et al. produced an independent antibody against ARV7 and detected the 75kDa ARV7 protein in C-81, C4-2, C4-2B, CWR-R1, and 22Rv1 cells [128]. This was the second study to show that ARV7 could not be detected at the protein level in androgen-responsive LNCaP cells [128]. Considering that recurrent cell lines derived from LNCaP, C-81, C4-2, and C4-2B, did express ARV7 protein, these data suggest that increasing levels of ARV7 could be a mechanism by which these cells become resistant to androgen deprivation [127, 128].

**Table 4:** Androgen receptor splice variants

Variant	Protein Regions	Activity	References
ARV7 (AR3)	NTD, DBD	Ligand-independent, Nuclear	[119–120,122–123, 126]
AR^v567es^	NTD, DBD, Hinge	Ligand-independent, Nuclear	[121, 126]
ARV12	NTD, DBD, Hinge	Ligand-independent, Nuclear	[123]
AR^1/2/2b^ (ARV3)	NTD, partial DBD	Ligand-independent	[118–120]
AR^1/2/3/2b^ (ARV4, AR5)	NTD, DBD	Ligand-independent	[118–120]
ARV1 (AR4)	NTD, DBD	LNCaP: Ligand-independent, PC3: Inactive, Cytoplasmic	[119–120, 123,126]
ARV9	NTD, DBD	LNCaP: Ligand-independent, PC3: Inactive, Cytoplasmic	[123, 126]
ARV13	NTD, DBD, Hinge, partial LBD	Inactive	[123]
ARV2	NTD, DBD	Not determined	[119]
ARV5/V6	NTD, DBD	Not determined	[119]
ARV8/10/11	NTD, DBD	Not determined	[126]
ARV14	NTD, DBD, Hinge, partial LBD	Not determined	[123]

There is also evidence of AR variant transcripts derived from exon skipping. With primers against AR exon 2 and 8, RT-PCR using cDNA generated from LuCaP xenografts produced a short amplicon lacking exons 5, 6, and 7 [129]. ARv567es is considered to lack the LBD since it only contains 10 amino acids coded for by exon 8 and as with other AR splice variants that lack the LBD, ARv567es is constitutively active (Table 4) [129]. Exogenous expression models by several groups have shown that ARv567es is capable of being translated [129–133], but due to the nature of its structure (Figure 1C), a variant-specific antibody that recognizes endogenous ARv567es has yet to be produced [124, 125]. In another study, 3′ RACE using a primer anchored at the border on AR exons 2 and 3 followed by next-generation sequencing (NGS) identified 6 AR variants, including ARV1 and ARV7 and 4 novel variants termed ARV 8-11 (Table 4) [134]. The ARV9 transcript contained a new exon (CE5) and ARV8, ARV10, and ARV11 only differed from each other by a unique downstream 3′ sequence coded for by intron 3, but all lacked the LBD (Table 4) [134]. Finally, Hu et al. identified AR splice variants *via* an unbiased method with a protocol using *in vitro* transcription and not primer-directed PCR [131]. This method confirmed variants ARV7, ARv567es, AR1/2/2b, ARV1, ARV2, and ARV5/V6 (Table 4), but also identified AR transcripts containing 3′ regions that corresponded to sequences downstream of AR exon 8—this novel exon was named exon 9 [131]. Three new AR variants, named ARV 12-14, contained exon 9 and also lacked one or more exons that code for the LBD (Table 4) [131]. Of these exon skipping AR variants, ARV12 was identical to ARv567es except it contained exon 9 and ARV13 and ARV14 coded for partial LBDs (Table 4) [131]. Further analysis of ARV 12-14 showed that ARV12 was constitutively active and localized to the nucleus, as expected considering the known activity of ARv567es, and that ARV13 and ARV14 were inactive [131]. Based on these data, expression of ARV12 (ARv567es) likely contributes to development of CR-PCa and resistance to therapy, but involvement of ARV13 and ARV14 in these resistance mechanisms appears to be improbable.

### Activity of androgen receptor splice variants

Luciferase assays were utilized to characterize the activity of AR variants in many studies, but these data represent AR transcriptional activity at only exogenously-introduced promoters. When compared to the LNCaP cells treated with R1881, LNCaP cells expressing ARV7 had equivalent induction of 20 different androgen-regulated genes (ARGs); providing evidence of ARV7's constitutive activity in endogenous gene transcription [127]. Similarly, overexpression of ARv567es in LNCaP cells also activated expression of ARGs PSA, TMPRSS2, and FKBP5 [129]. Transcriptional profiles specifically induced by AR variants were further characterized by overexpressing AR-FL or ARV7 in LNCaP cells and performing comprehensive gene expression microarray analysis [135]. Data showed that gene sets enriched in ARV7-overexpressing cells, including genes involved in the cell cycle, were distinct from those enriched in AR-FL-overexpressing cells, including genes involved in biosynthesis, metabolism, and secretion (Figure 1D-1F) [135]. These data were novel since previous reports had suggested that the transcriptional activity of AR variants is dependent on the presence of AR-FL [129, 134]. This discrepancy was addressed by Cao et al. who used chromatin immunoprecipitation (ChIP), a method that immunoprecipitates a DNA-binding protein and subsequently analyzes the bound DNA by PCR, to analyze the co-occupancy of ARV7 and AR-FL at the promoters of PSA and UBE2C, a gene specifically regulated by ARV7 [133, 135]. In this study, ARV7 ChIP, re-ChIP for AR-FL, and quantitative PCR for the promoter regions of PSA showed that both ARV7 and AR-FL occupied the PSA promoter under basal conditions, in the presence of DHT, and following treatment with the second generation anti-androgen enzalutamide [133]. Conversely, the UBE2C promoter region was not amplified using the DNA material immunoprecipitated by ARV7 ChIP/AR-FL re-ChIP, suggesting that both ARV7 and AR-FL do not co-occupy this promoter [133]. These data suggest that ARV7 both induces constitutive activation of canonical ARGs by heterodimerizing with AR-FL and activates a unique set of target genes independent of AR-FL (Figure 1G) [133]. Altogether, the detailed experimentation described above demonstrates that AR variants play an active role in promoting development of CR-PCa by reestablishing expression of ARGs and inducing expression of their own set of target genes.

### Androgen receptor splice variant expression in clinical samples

Several studies have shown that AR splice variants are expressed in PCa tumor samples (Table 5). Semi-quantitative RT-PCR showed that ARV1 and ARV7 expression were significantly increased in CR-PCa primary and metastatic tumor specimens compared to hormone-naïve PCa or normal prostate tissue [127]. In hormone-naïve PCa specimens, relative expression of ARV7 correlated with disease recurrence, in that patients with ARV7 expression greater than median had decreased time to PSA recurrence following prostatectomy (Table 5) [127]. In comparison, ARV1 expression did not correlate with PSA recurrence in hormone-naïve patients (Table 5) [127]. Tissue distribution of ARV7, as measured by immunohistochemistry using tissue microarrays, was distinct in benign, hormone-naïve, and CR-PCa [128]. Quantitative score analysis showed that ARV7 staining was significantly increased in hormone-naïve tumors compared to benign tissue and in CR-PCa tumors compared to hormone-naïve tumors [128]; illustrating that ARV7 may be used as a biomarker. Expression analysis of ARV7 and ARv567es by quantitative RT-PCR in bone metastases collected from CR-PCa patients during orthopedic surgery showed that increased expression of either ARV7 or ARv567es was associated with decreased cancer-specific survival [136]. These data were the first to show that AR variants are associated with lethal CR-PCa [136]. Similarly, a recent study has shown that expression of ARV7 in circulating tumor cells (CTCs) correlated with therapy response and survival in metastatic CR-PCa patients receiving the second generation ADT agents enzalutamide or abiraterone (Table 2); in that, patients with ARV7-positive CTCs had lower PSA response rates and shorter median overall survival [137]. This significant correlation between ARV7 expression and failure of second generation ADT treatments has prompted a clinical trial to study the correlation of AR-V7 CTC assays with clinical progression in CR-PCa and determine its utility as a biomarker/device (NCT02269982, https://clinicaltrials.gov/). In addition, several clinical trials are already using ARV7 expression analysis in CTCs to stratify their CR-PCa patient populations and assess effectiveness of treatments, including cabazitaxel (Phase II, NCT02621190, https://clinicaltrials.gov/) and galaterone (Phase III, NCT02438007, https://clinicaltrials.gov/) based on ARV7 expression status. The results of the clinical trials aimed to assess ARV7 as a biomarker and determine the effectiveness of specific therapies in ARV7-expressing CR-PCa patients are awaited in anticipation to determine the true clinical utility of AR splice variants in governing patient decision-making.

**Table 5:** Androgen receptor splice variants identified in clinical samples

Variant	Treatment	Tissue Type	Relationship to Disease	References
ARV7 (AR3)	Orchiectomy, LHRH, CAB, Enzalutamide	Primary, Metastases: Lymph node, Bone, Liver, Adrenal, Soft tissue, CTCs	Correlates with PSA recurrence after prostatectomy, cancer-specific survival, overall survival	[119–121, 129]
AR^v567es^	Orchiectomy, LHRH, CAB	Primary, Metastases: Lymph node, Bone, Liver, Lung	Correlates with cancer-specific survival	[121, 128]
ARV1 (AR4)	Orchiectomy, LHRH, CAB	Primary, Metastases: Lymph node, Bone, Liver, Adrenal, Soft tissue	No correlation	[119–120, 123]

## RESISTANCE MECHANISMS NOT RELATED TO ANDROGEN RECEPTOR

While understanding the role of androgen signaling and AR activity has been at the forefront of research identifying molecular drivers of CR-PCa, there are also mechanisms that possibly govern resistance to ADT that do not directly involve androgen signaling. For the purpose of this review article, we will limit our discussion of those pathways considered clinically relevant, including activation of glucocorticoid receptor (GR), impairment of DNA repair pathways, and expression of microRNAs.

### Glucocorticoids and glucocorticoid receptor

Multiple treatment regimens for PCa include glucocorticoid administration [65]. As discussed above, inhibiting the LHRH-LH axis with first-line ADT therapy does not efficiently reduce production of adrenal androgens [72]. To decrease plasma levels of DHEA, glucocorticoids have been used as a means to suppress CRH-ACTH during primary ADT [65]. Inhibition of CYP17A1 by abiraterone in second-line ADT hinders the production of androgens, oestrogens, and glucocorticoids, but not mineralcorticoids [65]. Therefore, perturbation of steroid synthesis by abiraterone leads to excess production of mineralcorticoids that promote increased blood pressure and potassium loss [65]. In addition, glucocorticoid deficiency halts the negative feedback loop with the hypothalamus/pituitary, activates the CRH-ACTH signaling axis, and increases production of adrenal androgens [65]. During initial clinical trials, the side effects caused by abiraterone as a result of altered steroid biosynthesis necessitated co-treatment with the glucocorticoid prednisone to control these adverse effects (Table 2) [56, 57, 138, 139]. Finally, both chemotherapeutic treatments FDA-approved for CR-PCa, docetaxel and cabazitaxel, are also administered with prednisone (Table 2) [60–63].

It has been known since the early 2000s that glucocorticoids promote PCa cell proliferation. Treatment with either cortisol, the primary glucocorticoid in circulation, or cortisone, a metabolite of cortisol, increased proliferation of the recurrent MDA-PCa 2b cell line [112]. In contrast, neither cortisol nor cortisone affected proliferation of the androgen-sensitive LNCaP cell line [112]. Both cortisol and cortisone increased PSA release by MDA-PCa 2b cells into the cell culture media, which was not observed for LNCaP cells [112]. The primary conclusion of this study was that the increased proliferation and PSA release was the direct result of expression of the glucocorticoid-responsive AR L702H mutant in MDA-PCa2b cells and not in LNCaP cells (Table 3) [112]. However, the authors of this study also showed that glucocorticoid receptor (GR), a nuclear receptor part of the same steroid receptor family as AR [140], is expressed in MDA-PCa 2b cells by immunoblot [112]. Therefore, it is unclear whether activation of GR in the recurrent cell line MDA-PCa 2b could be responsible, at least in part, for the growth stimulation induced by glucocorticoids. Nevertheless, these data suggest that glucocorticoid treatment of PCa patients during ADT or chemotherapy could possibly promote continued growth of PCa cells.

The amino acid sequences of the DBDs of AR and GR are 80% homologous [141]; therefore, substantial overlap of DNA-binding and transcriptional activities is present for AR and GR [65, 142]. Specifically in PCa, when LNCaP cells that stably express GR were treated with the corticosteroid dexamethasone and DNA binding of AR or GR was assessed by ChIP followed by NGS (ChIP-seq), it was found that 50% of the AR cistrome overlaps with the GR cistrome [142]. In the recurrent VCaP cell line that expresses endogenous GR, ChIP-seq showed that AR and GR cistromes overlapped by 58% [142]—suggesting that increased occupation of AR DNA binding sites by GR could contribute to castration resistance. Indeed, mRNA and protein expression of GR is increased in AR-overexpressing LNCaP xenografts resistant to enzalutamide [143]. The authors of this study established a cell line from the enzalumide-resistant AR-overexpressing LNCaP xenograft, selecting for high GR expression [143]. When tumor xenografts were established with this GR-overexpressing cell line in the presence of enzalutamide, these tumors were capable of immediate growth, while the parental cell line was not—showing that this cell line maintains it resistance phenotype [143]. Knockdown of GR prior to implantation inhibited xenograft tumor growth, establishing that GR expression is required for survival of this LNCaP AR/GR-overexpressing cell line in the presence of enzalutamide [143]. ChIP-seq data showed that when enzalutamide-resistant AR/GR-overexpressing LNCaP cells were treated with DHT, 52% of the AR binding sites were also occupied by GR [143]; confirming data from other groups using traditional PCa cell lines [142]. These data show that not only is GR increased in expression during the progression of enzalutamide resistance, but that GR activity is also activated. Arora et al. went on to assess GR expression by immunohistochemistry in matched bone marrow biopsy specimens from CR-PCa patients prior to and 8 weeks after starting treatment with enzalutamide [143]. In patients who were non-responsive to enzalutamide, there was a significant increase in number of GR positive cells both compared to baseline and to responsive patients [143]. Altogether, the data described above show that glucocorticoid signaling through the activation of GR is one of the mechanisms by which PCa becomes resistant to primary ADT by virtue of GR's ability to bind to AREs. In addition, increased GR expression and activity may be a mechanism governing continued survival of CR-PCa during second-line ADT with enzalutamide. Finally, the use of glucocorticoids to supplement ADT and chemotherapy remains a concern in the field [65]. Consequently, a clinical trial is ongoing to assess abiraterone with alternative doses of and treatment schedules for prednisone and alternative glucocorticoids (dexamethasone) in metastatic CR-PCa patients who are chemotherapy-naïve (Phase II, NCT01867710, https://clinicaltrials.gov/). It is the hope that a different glucocorticoid regimen will be able to suppress the side effects of abiraterone, but also limit a patient's exposure to these steroids that enable survival of PCa cells in an androgen-deprived environment.

### Impairment of DNA repair pathways

It has been known since the mid-1990s that genomic abnormalities are associated with PCa [144–146]. Specifically, early studies of primary PCa found chromosomal loss and instability at microsatellites, regions of the genome containing repeats of one nucleotide or groups of up to 5 [144–146]. The DNA repair pathway studied in early follow up investigations was mismatch repair [147–149], since this pathway is involved in correcting mistakes resulting from slippage at microsatellites during DNA replication [150]. In addition, loss of mismatch repair function has been associated with microsatellite instability [150]. When expression of proteins involved in mismatch repair, including MLH1, MSH2, MSH6, PMS1, and PMS2, were assessed by immunoblot in a panel of PCa cell lines, several cell lines had decreased expression of one or more of these proteins compared to HeLa cells that have normal mismatch repair function [147]. For instance, LNCaP had decreased levels of MSH2 and 6, DU145 has decreased levels of MLH1 and PMS1 and 2, and PC3 had decreased levels of PMS2 [147]. These data confirmed an earlier study that showed that MSH2 is undetectable in LNCaP; indeed, it was shown that LNCaP cells have homozygous deletion of exons 9-16 of MSH2 which results in a truncated protein [149]. In addition, it has been shown that DU145 cells harbor a mutation in the splice acceptor site between introns 1 and 2 of MLH1 that causes a frameshift mutation and premature stop codon [147]. Altered mismatch repair protein expression and mutations in these cells lines leads to deficient mismatch repair activity, where an *in vitro* assay showed that microsatellite instability was increased in DU145 and PC3 cells compared to HeLa cells [147]. Together, these experiments in cell lines show that alterations in DNA mismatch repair could be involved in PCa pathogenesis. When immunohistochemistry for MLH1, MSH2, and PMS2 was performed on primary tumor samples from PCa patients, loss or reduction of MSH2 or PMS2 significantly correlated with Gleason Score ≥ 4 in the entire patient cohort [148]. Specifically in patients of African American descent, known to have increased incidence and severity of PCa [151], loss or reduction of MLH1 significantly correlated with Gleason Score ≥ 4 [148]. These data suggest that insufficient expression of mismatch repair proteins, and presumably diminished mismatch repair activity, promotes the development of more advanced PCa.

In addition to playing a role in initial development of PCa, DNA repair pathways in PCa tissues are inhibited by ADT. Al-Ubaidi et al. performed prostate biopsies on PCa patients following initial diagnosis and after short-term ADT (1 month after bilateral orchidectomy and 2 months after initiation of LHRH agonist) to assess whether castration affects the activity of non-homologous end-joining (NHEJ), one pathway responsible for repairing double stand breaks (DSBs) [152]. Their hypothesis that ADT interferes with NHEJ originated from their data that showed that AR interacts with Ku70, a protein that binds DSBs and initiates NHEJ [153], in PCa tumor tissue [152]. Indeed, earlier studies also showed that AR and Ku70 interact in LNCaP cells [154]. Together, these co-immunoprecipitation studies in PCa cells and biopsies suggest that interfering with AR activity *via* ADT may impair the activity of Ku70. Al-Ubaidi et al. performed immunohistofluorescence for Ku70 before and after castration from the same tumor, which showed that Ku70 nuclear staining was decreased in castrated tissue [152]. When these imaging data were quantified, Ku70 nuclear staining intensity was significantly decreased by 50% in castrated PCa tumor tissue compared to staining intensity in matched tissue prior to castration [152]. To determine if decreased Ku70 as a result of ADT increases unrepaired DSBs, matched tumor biopsies were stained for γ-H2AX [152], a histone mark localized to DSBs [155]. Quantified immunohistofluorescence data showed that there was a significant correlation between decreased Ku70 and increased γ-H2AX in castrated PCa tumor tissue [152]. Together these data show that ADT impairs NHEJ, promoting persistence of unrepaired DNA damage in PCa tumors.

The intent of the research described above was to explain why outcomes for PCa patients receiving ADT and radiotherapy were better than those receiving radiotherapy alone [152]. In addition, it was the hope that establishing how castration increases sensitivity of PCa to radiotherapy would determine the best timing of ADT in this treatment regimen. A follow-up study by the same research group showed that patients who received an LHRH agonist for 8 weeks prior to radiotherapy had decreased Ku70 and increased γ-H2AX staining intensity compared to patients who received radiotherapy first followed by 8 weeks of LHRH agonist [156]. Another study determined that genes involved in multiple DNA repair pathways, including base excision repair, mismatch repair, homologous recombination, and NHEJ, are direct AR target genes—suggesting that inhibition of AR activity by ADT could have an even more widespread negative effect on the DNA damage response [157]. Together, these data suggest that impairment of DNA repair by ADT is beneficial for patients with intermediate- and high-risk PCa (Table 1) by increasing the amount of unresolved DNA damage and thus sensitizing PCa cells to radiotherapy [156, 157].

While deficiency in the DNA repair response is advantageous during PCa radiotherapy, data suggests that long-term impairment of DNA repair pathways by ADT may contribute to resistance in advanced/metastatic PCa. Genomic alterations in genes associated with the DNA damage response have been found in 22.7% of metastatic CR-PCa samples [115]. These genes include those involved in homologous recombination (BRCA1, BRCA2, RAD51B, and RAD51C), mismatch repair (MLH1 and MSH2), and DSB repair (ATM) [115]. The majority of alterations in these genes were deletions or frameshift mutations, suggesting that loss of these genes promotes development of CR-PCa [115]. Nevertheless, as with radiotherapy for primary PCa, the impaired DNA damage response in CR-PCa has been exploited to improve therapeutic outcomes. In a phase II trial, patients who had recurred after at least two rounds of CR-PCa therapies (Table 2) were treated with olaparib, an inhibitor of the base excision repair protein poly(adenosine diphosphate [ADP]-ribose) polymerase (PARP) [158]. Results showed that patients with genomic defects in DNA repair genes (e.g. BRCA1, BRCA2, ATM) had a 6.3 month increase in overall survival compared to those who did not have these defects [158]. These data illustrate that while impairment of the DNA damage response by ADT may promote progression of CR-PCa, genomic instability can make select CR-PCa more responsive to drugs that target DNA repair [158]. These results led the FDA to grant olaparib a Breakthrough Therapy Designation in 2016 for metastatic CR-PCa patients who have somatic or germline mutations in BRCA1, BRCA2, or ATM since this patient population failed to respond to approved therapies (Table 2). However, this phase II trial included only 50 patients, 14 (28%) of which had DNA repair gene mutations and responded to olaparib [158]; therefore olaparib is presumably effective in less than one-third of the metastatic CR-PCa patient population, granted that the percentage of responders remains consistent in a larger cohort. In addition, all of the CR-PCa patients in this trial had recurred with docetaxel and 80% had received ≥ 4 treatment regimens for CR-PCa [158]. Therefore, even the limited number of CR-PCa cases that may be sensitive to chemical inhibition of DNA repair are from a population of the most advanced cases. It is our opinion that research efforts should be focused on understanding more universal mechanisms of ADT resistance to prevent patients from ever reaching such advanced CR-PCa disease.

### Expression of microRNAs

MicroRNAs (miRs) are endogenously expressed short non-coding RNA molecules that negatively regulate gene expression by binding to mRNAs and inhibiting translation [159]. A comprehensive analysis of miR expression in BPH, hormone-naïve PCa, and CR-PCa samples performed in 2007 using oligonucleotide array hybridization showed that 51 miRs were differentially expressed in cancerous lesions compared to benign tissue [160]. These data indicate that altered expression of miRs correlate with initial PCa pathogenesis [160]. Thirty-seven miRs were decreased in both hormone-naïve and CR-PCa samples, whereas 15 miRs were decreased in CR-PCa only; showing that hormone-naïve and CR-PCa tumors have distinct miR expression patterns [160]. A more recent study using a miR microarray found that miR expression was more divergent between primary and CR-PCa tumors, where 75 miRs were differentially expressed in primary PCa, 88 miRs were differentially expressed in CR-PCa, and changes in expression for only 22 miRs overlapped between primary and CR-PCa samples [161]. Together these data suggest that changes in miR expression could contribute to resistance to ADT.

The study of miRs in PCa pathogenesis and development of CR-PCa has shown that specific miRs can act either as tumor suppressors or oncogenes, where tumor suppressor-miRs are decreased and oncogenic-miRs are increased in expression [162, 163]. The most well-studied tumor suppressor-miRs that contribute to PCa progression include miR-15/16, miR-34, miR-143, miR-200c, and miR-101 [162–172]. Studies in cell lines show that exogenous expression of miR-16 inhibits growth of recurrent 22Rv1, DU145, and PC3 cells, but not of androgen-dependent LNCaP cells [164]. Similarly, endogenous expression of miR-34a is decreased in DU145 and PC3, but unchanged in LNCaP compared to normal prostatic epithelial cells [167]. miR-34a has been shown to specifically downregulate expression of AR [165], suggesting that loss of miR-34a could contribute to increases in AR expression observed in human CR-PCa tumors, cell lines, and xenograft models described above [91, 92]. Finally, when miR-34b expression was stratified as low, medium, or high in PCa patient samples, low miR-34b expression correlated with Gleason score 8-10 and high miR-34b expression correlated with longer overall survival, suggesting that decreased miR-34b expression correlates with aggressiveness of primary PCa [168]. These data indicate that miRs not only act mechanistically to promote PCa progression and resistance to ADT, but can also act as biomarkers for advanced disease [163]. Similarly, expression of miR-143 inversely correlated with Gleason score of primary PCa [169], miR-200c expression was significantly decreased in primary tumors from patients who relapsed following prostatectomy compared to those who did not relapse [170], and miR-101 expression was significantly decreased in metastatic compared to primary PCa samples [171]. Oncogenic-miRs that have been shown to promote PCa resistance to ADT include miR-221/222 and miR-21 [162, 163, 172–174]. Expression of miR-221/222 was found to be significantly increased in CR-PCa, while significantly decreased in primary tumors [172]. These data suggest that the activation of miR-221/222 promotes resistance to ADT [172]. Finally, expression of miR-21, an oncogenic-miR that is most commonly overexpressed in solid tumors [163], also correlates with PCa aggressiveness [173, 174]. Increased miR-21 levels in serum significantly correlated with increased PSA in patients undergoing ADT and those that were castration resistant [174]. Expression of miR-21 at the tissue level was also positively correlated with Gleason score > 7 and patients with miR-21 negative tumors had increased biochemical recurrence-free survival [173]. Overall, multiple miRs play a role in initial PCa pathogenesis, progression, and development of CR-PCa. In addition, it is possible that expression of individual miRs may act as biomarkers for identifying those patients most likely to develop ADT resistant disease. For detailed analysis of individual miRs and their target genes relevant to PCa, readers are directed to recent review articles by Ayub et al. [162] and Kojima et al. [163].

## RESISTANCE TO SECOND GENERATION ANDROGEN DEPRIVATION THERAPY

Abiraterone and enzalutamide are recently approved therapies for CR-PCa that target androgen signaling (Table 2), the mechanisms of action of which have been lately reviewed by our group [33]. These novel treatments were hailed at the time for increasing overall survival of CR-PCa patients to a greater extent than the chemotherapeutic agent docetaxel (Table 2) [56–59]. However, 20%-40% of the patients receiving treatment in these studies are non-responders, which ranged based on drug and previous treatments [56–59, 175]. In addition, even those patients who initially respond to abiraterone or enzaluamide all develop resistance [175]. There is evidence to suggest there are pathways of resistance specific to these second generation ADT agents, but considering these therapies were approved in the 2010s, a comprehensive body of work has yet accumulated at the time of this writing. In this section, we will discuss studies that have addressed specific mechanisms governing resistance to either abiraterone or enzalutamide.

Abiraterone targets androgen biosynthesis by inhibiting CYP17A1, the role of which is to convert pregnenolone to 17α-OH-pregnenolone and 17α-OH-pregnenolone to DHEA during steroidogenesis [78]. Cai et al. established VCaP xenografts and mice were treated with abiraterone (0.1 mg/mL in drinking water) until relapse, defined as restored tumor growth that occurred between 4-6 weeks of treatment [78]. When compared to before abiraterone treatment, CYP17A1 mRNA expression was increased in abiraterone-resistant VCaP xenografts [78]. In contrast, VCaP xenografts harvested after only 8 days of abiraterone (0.5 mg/day every other day by intraperitoneal injection) showed no change in CYP17A1 mRNA expression compared to pre-treatment tumor samples [78]. These data suggest that long-term treatment with abiraterone, but not short-term, promotes a resistant tumor environment where increased CYP17A1 expression possibly compensates for CYP17A1 inhibition by abiraterone [78]. This study also showed that AR activity was not suppressed by abiraterone in the LNCaP-derived recurrent C4-2 cell line, suggesting that these cells display primary resistance to abiraterone [78]. As discussed above, the progesterone-responsive AR T878A mutant is expressed in cells of LNCaP lineage (Table 3) [102, 176]. Since progesterone is generated from cholesterol *via* the enzymes CYP11A1 and HSD3B1 or 2, not CYP17A1, it was postulated that survival of C4-2 cells with abiraterone is dependent on expression of AR T878A [78]. This hypothesis was the subject of a study by Chen et al. that aimed to identify if AR T878A was expressed in patients who develop resistance to abiraterone [177]. A specific cDNA pool was generated with a primer against the 3′-UTR region of AR using metastatic bone marrow or soft tissue biopsies from CR-PCa patients who progressed on abiraterone [177]. Nested PCR was then performed to amplify the AR LBD with primers against the 3′-UTR and DBD and reactions were submitted to NGS [177]. The number of sequencing reads with the AR T878A mutant were compiled, showing that AR T878A was expressed at a high allele frequency in 2 out of 17 (11.8%) abiraterone-resistant patients [177]. These data suggest that maintenance of tumor growth in these patients could be the result of AR T878A expression [177]. Interestingly, one of these AR T878A-expressing patients took abiraterone for 40 months, the longest in the cohort, and his initial response was the most robust at 99% reduction in PSA [177]. The second patient who expressed AR T878A took abiraterone for only 8 months and had an initial PSA response of 68% [177]. Finally the only patient in this cohort who showed no response to abiraterone did not express AR T878A [177]. Altogether, while maintenance of AR signaling in patients who express AR T878A possibly contributes to abiraterone resistance, it is unclear from this small patient cohort if this mechanism truly drives resistance or if these data are the result of coincidental sequencing results.

As discussed in detail above, several lines of evidence suggest that resistance to enzalutamide may be governed by increased GR expression and GR-dependent activation of ARGs [65]. Enzalutamide-specific resistance has also been linked to expression of a novel AR mutant [55, 178]. Joseph et al. derived resistant LNCaP and LNCaP-AR overexpressing cell lines by prolonged treatment with either enzalutamide or ARN-509 [178], another AR antagonist similar to enzalutamide [33]. In 3 out the 6 resistant cell lines, including 2 resistant to ARN-509 and 1 resistant to enzalutamide, an AR missense mutation was discovered that leads to an amino acid change from phenylalanine (F) to leucine (L) at position 877 (F877L) (Table 3) [178]. In addition, these cell lines also contained the T878A mutation (Table 3) [178]. Instead of inhibiting growth in these resistant cell lines, enzalutamide or ARN-509 treatment promoted cell proliferation [178]. When AR F877L was overexpressed in the parental cell lines, enzalutamide or ARN-509 induced cell proliferation, promoted AR F877L binding to DNA, and activated expression of ARGs [178]. When xenografts were established using an LNCaP cell line stably overexpressing AR F877L, neither enzalutamide nor ARN-509 affected tumor growth [178]. Together these *in vitro* and preclinical data suggest that AR F877L contributes to enzalutamide resistance *via* its ability to be activated, rather than inhibited, by this second generation ADT drug [178]. In a recent study, NGS for AR exon 8 using circulating free DNA detected AR F877L in a patient that acquired resistance to both enzalutamide and abiraterone [116]; however, expression of AR H874Y and T878A mutants were also discovered in this patient. While the authors suggest that AR F877L contributes to enzalutamide resistance, there is no way to be sure whether AR F877L was responsible, if the other mutants were responsible, if the combination of AR mutants was responsible, or if another unknown mechanism could have led to progression in the patient. Together, the data collected thus far in studying resistance to abiraterone or enzalutamide in CR-PCa have yet to implicate a consistent mechanism for either drug that contributes to treatment failure. However, these data do suggest that persistence of androgen and AR signaling remains a hurtle to curative therapy.

## PRECISION MEDICINE IN PROSTATE CANCER THERAPY: DREAMS, PROMISES AND REALITIES

In this review article, we describe the mechanisms by which advanced/metastatic PCa becomes resistant to approved therapies, giving special attention to the molecular biology methods used to study this continuing challenge in urologic oncology. In the era of high-thoroughput DNA sequencing, many researchers and clinicians in the field have postulated that “personalized medicine” may achieve curative therapy for metastatic PCa. Personalized medicine is a hypothesis derived from the idea that molecularly-targeted therapies based on a patient's specific genetic or molecular profile will achieve more affective clinical outcomes [179, 180]. Indeed, improvement in experimental techniques and technology over the last several decades has contributed to a greater understanding of the detailed molecular mechanisms driving carcinogenesis in specific organ systems [179, 180]. While an attractive concept for scientists, clinicians, patients, politicians, the media and lay public, precision oncology for any tumor-type has yet to be empirically validated [179]. For example, the study described above showing that olaparib increased survival in CR-PCa patients specifically harboring mutations in DNA repair genes has been considered a success for precision medicine in PCa [158, 181–183], but has yet to be validated in a wider patient cohort. The ability to actually improve patient treatment *via* personalized oncology rests in the identification of biomarkers or gene panels that are biological relevant and development of clinical tests that are technically feasible in a routine setting. Obtaining sufficient material to perform genomic analysis and standardization of procedures both remain dilemmas in the field of personalized oncology [184]. The use of CTCs or circulating free DNA has been posited as an alternative to performing additional biopsies to obtain sufficient material to perform genomic analysis, especially in PCa [185]. Even as sequencing using circulating material becomes possible, as shown by the studies we have described in this article that detected ARV7 in CTCs [137] and AR LBD mutants in free circulating DNA [116], verification in larger patient cohorts and standardization of procedures must be developed [185]. In addition, analysis of all possible clinical interpretation of results must be carefully addressed [179]. Altogether, the feasibility of precision oncology for PCa, and other tumor types, remains in question due to lack of empirical validation and evidence that “precision” treatment approaches are superior to established therapies [179]. We believe that continued study of the universal molecular biology of CR-PCa, the approach taken by most of the studies described in this article, has more potential to develop curative therapy for a larger proportion of patients.

## Terminology and Abbreviations

ACTH: Adrenocorticotropic hormone
ADT: Androgen deprivation therapy
AR: Androgen receptor
ARE: Androgen response element
ARFL: Androgen receptor full length
ARG: Androgen-regulated gene
ARV: Androgen receptor variant
BLAST: Basic Local Alignment Search Tool
BPH: Benign prostatic hyperplasia
CAB: Combined androgen blockade
CE: Cryptic exon
ChIP: Chromatin immunoprecipitation
CRH: Corticotropin-releasing hormone
CR-PCa: Castrate-recurrent prostate cancer
CYP17: Cytochrome P450 17
DHEA: Dehydroepiandrosterone
DHT: Dihydrotestosterone
DRE: Digital rectal examination
DSB: Double strand breaks
EGF: Epidermal growth factor
FISH: Fluorescence *in situ* hybridization
FKBP51: FK506 Binding Protein 51
HSP: Heat shock proteins
IGF1: Insulin-like growth factor-1
KLK: Kallikrein-related peptidase
LBD: Androgen receptor ligand-binding domain
LHRH: Luteinizing hormone releasing hormone
MAPK: Mitogen-activated protein kinase
miRs: microRNAs
NGS: Next-generation sequencing
NHEJ: Non-homologous end joining
PCa: Prostate cancer
PKC: Protein kinase C
PI3K: Phosphoinositide 3-kinase
PSA: Prostate specific antigen
RACE: Rapid amplification of cDNA ends
RT-PCR: Real-time PCR
TMPRSS2: Transmembrane protease, serine 2
